# Analysis of the Impact of Graphite Addition on the Tribological Properties of Composites with Polyester–Glass Recyclate

**DOI:** 10.3390/ma18020376

**Published:** 2025-01-15

**Authors:** Grzegorz Hajdukiewicz, Aleksander I. Komarov, Dmitry V. Orda, Katarzyna Panasiuk

**Affiliations:** 1 Faculty of Marine Engineering, Gdynia Maritime University, 81-225 Gdynia, Poland; g.hajdukiewicz@wm.umg.edu.pl; 2 Joint Institute of Mechanical Engineering of the National Academy of Sciences of Belarus, The State Scientific Institution, 220072 Minsk, Belarus; al_kom@tut.by (A.I.K.); dmitry_orda@mail.ru (D.V.O.)

**Keywords:** graphite, recycling, composite materials, friction coefficient, composite structures

## Abstract

Composites are increasingly being modified with various types of fillers and nanofillers. These materials have attracted much attention due to the improvement in their properties compared to traditional composite materials. In the case of advanced technologies, adding additives to the matrix has created a number of possibilities for use in many industries, from electronics to mechanics. Mechanical recycling of composites allows them to be reused as a filler in new composite materials; however, a decrease in their strength parameters is observed, and hence, new possibilities of their use are sought. The main objective of this research was to analyze the effect of the graphite content on the tribological and structural properties of composites with polyester–glass recyclate. Composite materials with 10% polyester–glass recyclate and an additive in the form of graphite in the amounts of 0%, 2%, 5%, 10% were made using the hand lamination method and their mechanical properties were verified. Then, using a universal tribometer operating in rotational motion with friction without lubrication, the influence of nanoadditives on the change in the coefficient of friction (µ) and the change in the coefficient K (the rate of a mass wear) of the obtained composites was investigated. This study showed that adding graphite in the amount of 2%, 5%, and 10% changes the nature of tribological wear of the obtained composite material. The coefficient K (mass wear rate) also changes. The addition of 10% graphite significantly changes the coefficient of friction without lubrication in a pair with a steel counter-sample.

## 1. Introduction

Polyester–glass composites are materials that are used in various industries, including the aviation, automotive, shipbuilding, and railway industries [1,2,3]. Their widespread use requires finding methods for their recycling [4,5,6,7] and then for their application [8]. These issues concern not only the broadly understood wind energy and blade recycling [9], but also the yacht industry and others [10,11,12]. In [13], the mechanical properties of composites with polyester–glass recyclate were verified. The research results showed that with the increase in the percent content of recyclate ranging from 0 to 30%, a decrease in these properties was observed.

In the technology of composite materials, the type and distribution of the reinforcing phase in the composite matrix are of great importance. In order to improve the properties of composites in relation to conventional materials, it is necessary to ensure the uniform distribution of all components in the material structure, as well as their appropriate mutual bonding. The quality of this connection has a direct impact on the properties of the designed materials [14,15].

Regardless of the form, the addition of carbon has a positive effect in reducing the coefficient of friction. In [16], the lowest coefficient of friction μ, equal to 0.35, was obtained for the Cu–C composite with graphite nanopowder. The coefficient of friction for the remaining composites in the steady state was essentially identical and amounted to about 0.4. The form of carbon had a significant effect on the initial increase in the value of the coefficient of friction. The size of the fraction of the carbon nanoadditive and the uniformity of its distribution were the main factors determining the value of the coefficient of friction. Mechanical properties such as tensile strength and impact strength, as well as the modulus of elasticity of the composites with the addition of graphite, were significantly higher than without its addition. Further, hybridization with silicon carbide and graphite additionally improved the functional properties, as well as the thermal resistance of the composites [17].

The authors of [18] mainly described graphite and its various modification possibilities for use as modified fillers in polymer matrices for forming polymer–carbon nanocomposites. In [19], three sets of exfoliated graphite-filled polymers having three different particle sizes were prepared with loading levels ranging from 0.1 to 20 wt.%. The electrical, thermal, and mechanical properties of the nanoparticle-filled polymers were measured. Compared to the pure polymer, the polymers filled with 20 wt.% exfoliated graphite showed a significant reduction in electrical resistivity from 1.58 to 0.5 Ω cm. The thermal conductivity of the polymers containing 20 wt.% exfoliated graphite was also significantly improved, increasing from 0.2 to 5 W/mK. The flexural modulus reached a maximum increase of 3.8 GPa, which is 60% higher than the value for the pure polymer (2.4 GPa).

In [20], graphite-reinforced epoxy composites with different fractions of graphite particles were tested for their mechanical properties, such as tensile strength, impact strength, and bending strength. The graphite content varied from 2% to 8% by weight of the total matrix in the composites. The studies showed that the properties of the composites mainly depended on the dispersion state of the filler particles, the particle size. The composites showed higher tensile modulus, bending modulus, and impact strength in the case of bending properties with the increase in the filler content.

In [21], the impact of different amounts of graphite filler on the mechanical properties of epoxy composites reinforced with epoxy resin and carbon fiber was analyzed. Composites with graphite-reinforced epoxy matrix were prepared with graphite fractions ranging from 5 to 30 wt.%. Graphite–epoxy hybrids reinforced with carbon fiber were prepared using a constant amount of carbon fiber and introducing graphite into the epoxy in the amount of 7.5, 10, and 11.5 wt.%. After curing, the prepared materials were subjected to three-point tensile and bending tests. Increasing the content of the graphite filler had a direct effect in increasing the modulus of longitudinal elasticity. Materials with 7.5, 10, and 11.5 wt.% of graphite also showed an increase in the value of the ultimate stress with an increase in the filler. This study showed that carbon fiber reinforced graphite–epoxy composites exhibit better mechanical performance than conventional carbon fiber reinforced epoxy matrix composites.

In this paper, the effect of percent graphite content on the tribological properties of recycled polyester–glass composites was verified. Composite materials were made with 10% polyester–glass recyclate and a nanoadditive in the form of graphite in the following amounts: 0%, 2%, 5%, and 10%. They were then tested for mechanical [22] and tribological properties. The main goal was to verify whether the addition of graphite has an impact on improving the properties of composites with polyester–glass recyclate when they are used for structural elements.

## 2. Materials and Methods

**Figure 1:**
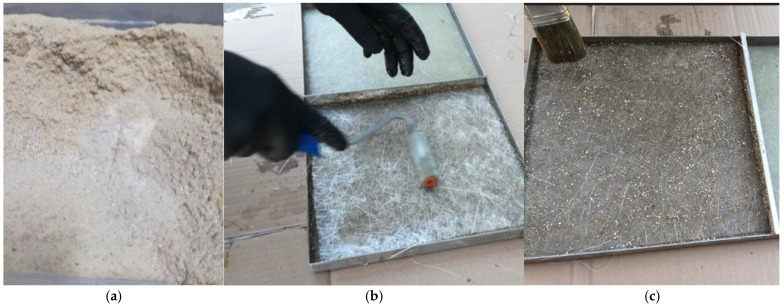
Composite with recyclate during the manufacturing process: (**a**) recyclate with a grammage of ≤1.2 mm; (**b**) percolation of subsequent composite layers; (**c**) finished composite with 10% recyclate content.

The first step was to produce composite materials in order to conduct structural, tribological, and mechanical tests [22]. Composite materials were produced using the hand lamination method. The manual lamination method is widely used in many industries, such as the yacht, automotive, rail transport, and aviation industries. Therefore, it is possible to implement the technology of manufacturing materials for the purposes of this article in the industry, and thus modify the process of infusion methods. The matrix was polyester resin, while the reinforcement was a glass mat with a random fiber direction (basis weight 350 g/m^2^). In total, 10% of recyclate with a fraction ≤1.2 mm was added to the resin. Also added was 0% (K10.0), 2% (K10.2), 5% (K10.5), and 10% (K10.10) of powdered graphite with a particle diameter of 0.044–0.074 mm by mass. In each case, 10 layers of reinforcement were used. The authors used polyester resin for manual lamination in the production process. Polyester laminating resin is an orthophthalic, thixotropic, and accelerated structural resin. The gel time of the selected resin at 25 °C is 19–26 min, with an exothermic peak of 75–115 °C, while the HDT (heat deflection temperature) is 63 °C. The glass transition temperature (Tg) of polyester resin is between 70 °C and 120 °C [22]. The polyester–glass recyclate was obtained by grinding polyester–glass laminates (fragment of the ship’s hull) by crushing and then grinding the waste until a fraction of ≤1.2 mm was obtained. Using the melting pot method, the fiber content in the composite was tested—40%. The recyclate was added to the resin and mixed with it, and then this mixture was used to percolate subsequent layers of the mat in the composite. In the material with the graphite, additional graphite was added to the mixture of recyclate and resin. Graphite powder is used to create external coatings that have a low coefficient of friction and an increased resistance to damage and scratches. Figure 1 shows the subsequent stages of manufacturing the composite material.

Table 1 presents the percent composition of the produced composite materials.

Table 2 presents the results obtained from strength tests using the static tensile test method.

The authors in [23] showed that adding 5% graphite powder to the matrix with 10% polyester glass recyclate content causes a slight decrease in mechanical properties, compared to the material without graphite powder. In the aforementioned studies, it was assumed that despite the decrease in strength parameters, it is possible to obtain a material with significantly higher tribological parameters.

Cylindrical samples with a diameter of 30 mm were prepared from the obtained composite material for tribological tests in friction without lubrication. Before the test, the samples were ground and polished to obtain horizontal friction surfaces. The grinding of the sample surfaces was performed mechanically with a stationary rotary grinder that had abrasive discs of different gradation. The final polishing of the surface was performed manually using diamond paste with a grain size of up to 3 μm. The surface roughness measurements of the samples after grinding and polishing were performed on an Olympus OLS40-SU confocal microscope (Tokyo, Japan) with Lext OLS4100 software version 3.1.7.14. The obtained surface roughness values were Ra in the range of 0.100 to 0.200 μm. Before weighing (performed to determine the mass wear rate K, a dimensionless proportionality constant that provides a measure of the wear intensity), loose material, which was a residue of grinding and polishing, was removed from each sample using an ultrasonic cleaner with distilled water.

**Figure 2:**
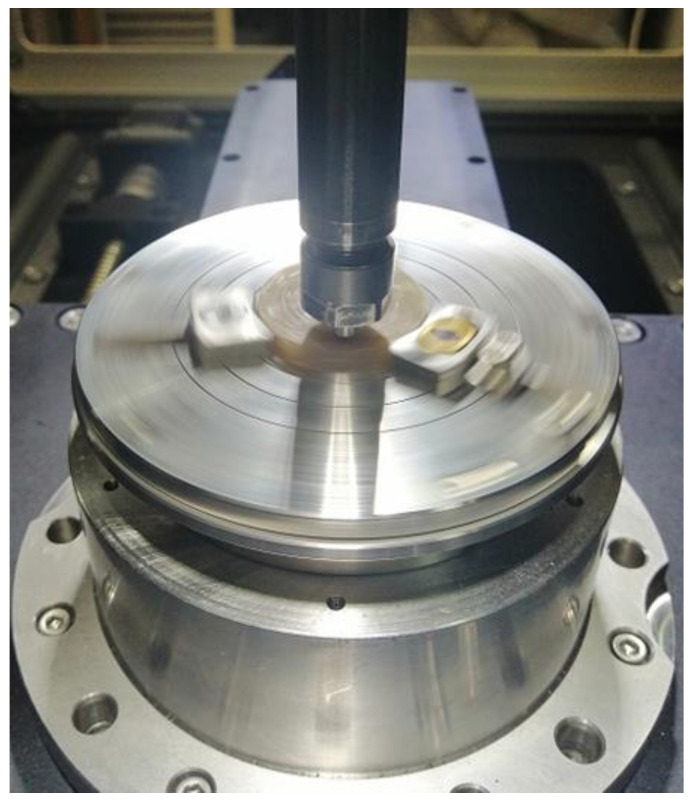
K10.0 composite sample during tribological test.

Tribological tests were carried out on an RTEC Instruments MFT-5000 tribometer (San Jose, CA, USA) in rotational motion (rotating sample and stationary counter-sample). The conditions in the room during the tribological tests were a temperature of 19°C and a relative air humidity of 48%. In the tribological test of all samples, the counter-sample was a cylinder with a chamfer that had a diameter of 6 mm made of 416 SS steel (X12Cr13) mounted in the tribometer mandrel. The cylinder was fixed as it had no possibility of rotating in the mandrel during the test. The rotational movement of the sample during the test is realized by the base on which the sample was mounted. This can be seen quite clearly in Figure 2. The counter-sample axis was offset from the sample rotation axis by 7.5 mm. The test was carried out without the use of lubricants, at a constant rotational speed of the sample of 120 rpm. The normal force N while loading the sample was 30 N. The duration of the test was 120 min for each sample, which was counted from the moment of reaching the set normal force N. The speed of force increase was programmed from 0 to 30 N in 300 s for each of the samples and each of the trials equally (0.1 N/s otherwise 6 N/min). The characteristics obtained during the test allowed the assessment of the effect of adding graphite to the polyester–glass composite with the addition of recyclate on, among others, the coefficient of friction μ and the coefficient K, the rate of mass wear. Figure 2 shows the K10.0 sample without the addition of graphite, during the test on the tribometer.

## 3. Results

**Figure 3:**
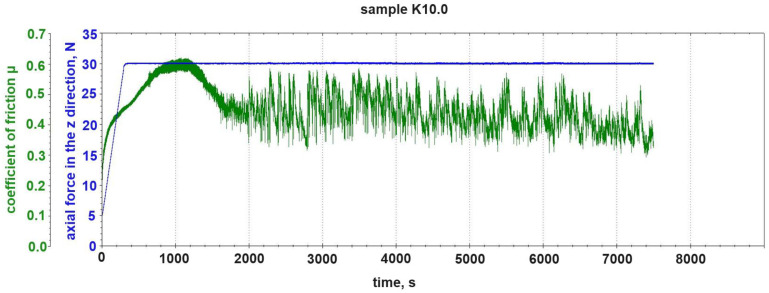
Change in friction coefficient μ and change in normal force load N during tribological test of sample K10.0 (without graphite addition).

The changes in the coefficient of friction during the tribological test were analyzed. Figure 3 shows the changes in the coefficient of friction μ during the tribological test of the K10.0 sample (without graphite addition). The figure also shows the load curve of the tested sample with the normal force N. The assumed and stable value of the normal force of 30 N until the end of the test was reached in the fifth minute of the test.

**Figure 4:**
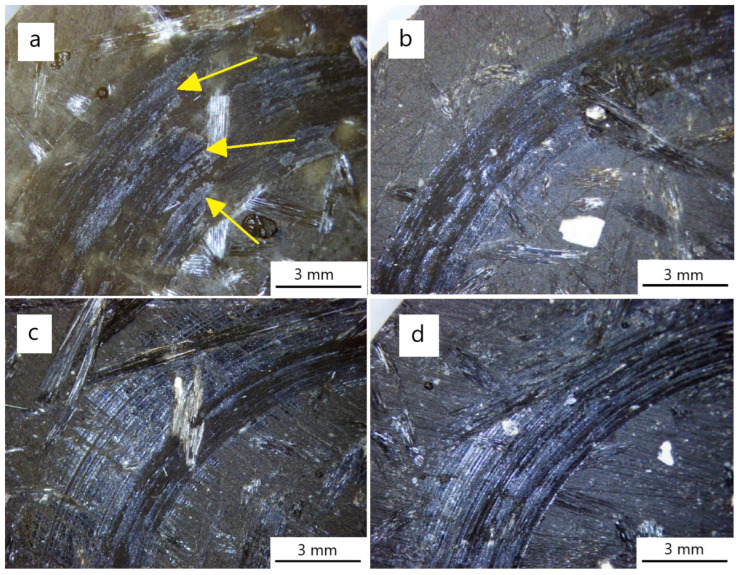
Surface structures after tribological testing of samples: (**a**) K10.0, (**b**) K10.2, (**c**) K10.5, and (**d**) K10.10.

Figure 4 shows again the course of the change in the friction coefficient μ of the K10.0 sample together with a graph of the change in the pin position in which the counter-sample was mounted during the tribological test. The change in the pin position was recorded along the z axis, i.e., the vertical axis along which the normal force N acting on the sample in the tribological test acts.

**Figure 5:**
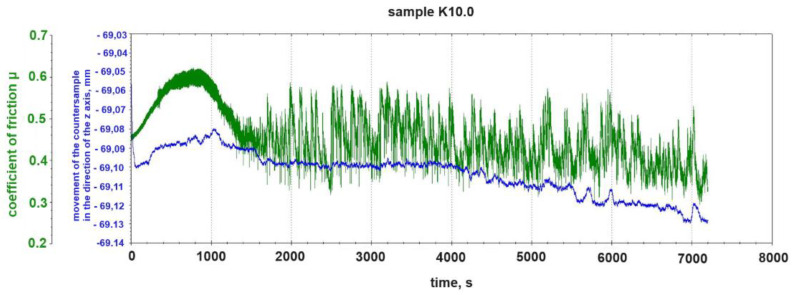
Change in friction coefficient μ and change in vertical position of counter-sample pin during tribological test of sample K10.0 (without graphite addition).

**Figure 6:**
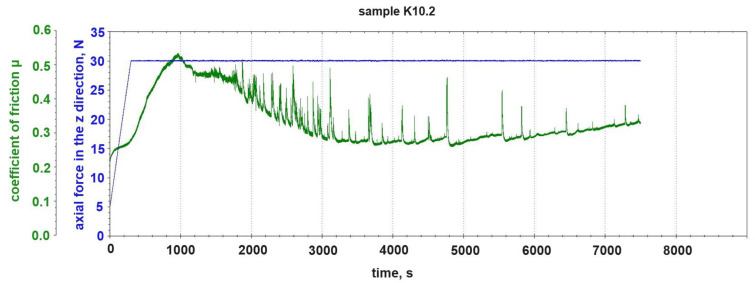
Change in friction coefficient μ and change in normal force load N during tribological test of sample K10.2 (2% graphite addition).

**Figure 7:**
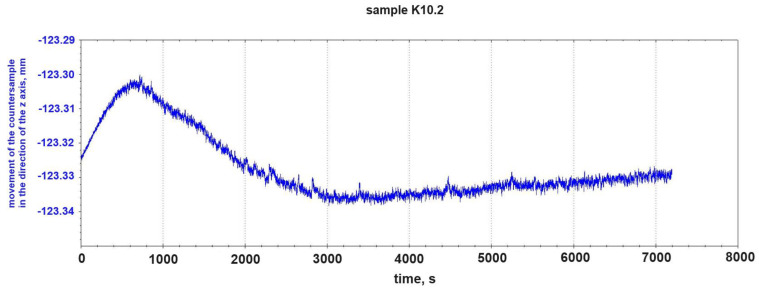
Change in vertical position of counter-sample pin during tribological test of sample K10.2 (2% graphite addition).

**Figure 8:**
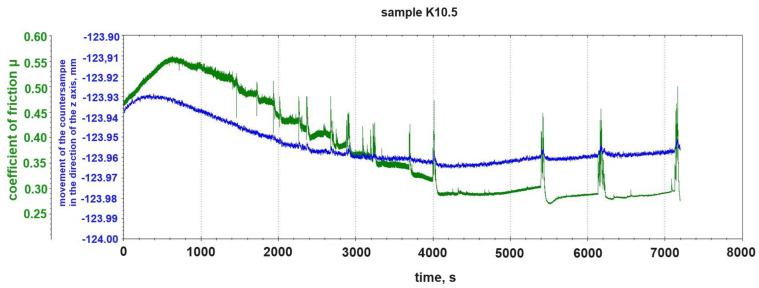
Change in friction coefficient μ and change in vertical position of counter-sample pin during tribological test of sample K10.5 (5% graphite addition).

**Figure 9:**
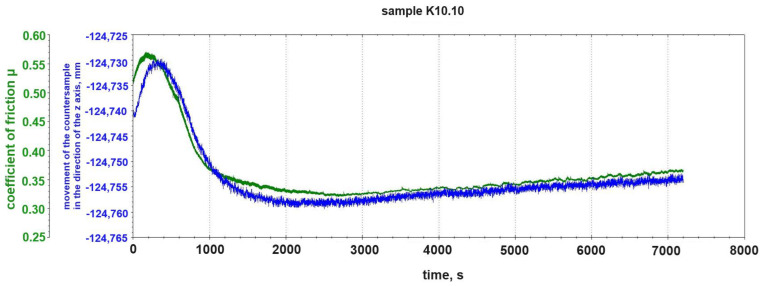
Change in friction coefficient μ and change in vertical position of counter-sample pin during tribological test of sample K10.10 (10% graphite addition).

Due to the much higher hardness of the steel counter-sample of 260 HB (Brinell hardness) and the stiffness of the pin and the pin-holder system in relation to the composite, the hardness was <60 HB [24]. The recorded change in the vertical position of the pin basically corresponds to the depth to which the counter-sample penetrated the composite structure. On the curve depicting the change in the coefficient of friction μ of the K10.0 sample during the tribological test, we distinguish the phase of the run-in of the sample–counter-sample pair. This phase ended after ~1400 s of the test, counting from the moment that the assumed value of the normal force N = 30 N was reached. The screenshots from the tribometer software (https://rtec-instruments.com/tribometer/universal-tribometer/?gad_source=1&gclid=Cj0KCQiAkJO8BhCGARIsAMkswyj42kmCAwWgAI8_7hktUq2mX-pY5sMl-xV1FvL31yFEgW294rj2IkcaAhGvEALw_wcB, accessed on 1 September 2024), which are Figure 5, Figure 6, Figure 7, Figure 8 and Figure 9, do not include the initial 300 s of the test in which the tribometer gradually loads the plunger with the counter-sample from 0 N to 30 N. According to the creators of the machine software, this phase of reaching the intended normal force should be discarded when determining the friction coefficient of the tested pair. The curve of the change in the coefficient of friction μ shows the mixed abrasive–adhesive nature of the tribological wear of the composite sample with the addition of the polyester–glass recyclate in friction with the steel counter-sample. Friction without the participation of lubricants of the steel counter-sample on the surface of the composite, with the addition of recyclate particles in the matrix, causes an increase in temperature on the friction surface. The increase in temperature causes the matrix (polyester resin) to transition from a solid state to a semi-liquid and liquid state. The recyclate particles and detached reinforcement, together with the liquid and semi-liquid matrix, cause the formation of accretions on the surface of the sample. Further movement during the test causes an increase in the friction coefficient and a further increase in the surface temperature until the build-up is torn off the sample. Then, the whole process is repeated. In Figure 4a, the arrows indicate the places on the surface of the K10.0 sample from which the build-up was torn off during the tribological test. These are denoted by characteristic broken, bright stripes on the friction path. The widths of the marks (friction paths) on the surface of the samples shown in Figure 4 are ~6 mm, which correspond to the diameter of the cylinder that was used as the counter-sample. The glass fibers of the base composite (0% graphite) that are visible in Figure 4 (especially Figure 4a) are the reinforcement of the material. The recyclate used to produce the material had a particle size of ≤1.2 mm. The reinforcement was exposed by grinding and polishing the surfaces during the preparation of the samples for tribological testing.

The alternating formation and separation of the build-ups from the sample is visible in the fluctuation of the vertical position of the tribometer pin (blue graph in Figure 5). A clear example of this phenomenon is seen in Figure 5 from 5400 s to 6000 s of testing.

Figure 6 shows the course of the change in the coefficient of friction μ during the tribological test of the K10.2 sample (2% graphite addition). The figure also shows the course of gradual loading of the tested sample with the normal force N. The assumed 30 N was reached in the fifth minute of the test. The course of the change in the coefficient of friction μ for the K10.2 sample differed quite significantly from the course of the change in the coefficient of friction μ for the base sample K10.0 without the addition of graphite. There was a clear decrease in the average value of the coefficient of friction μ and a clearly different course of the graph during the test. The addition of 2% graphite powder caused the dynamics of the mixed abrasive–adhesive nature of the tribological wear of the K10.2 sample to be significantly lower than in the case of the sample without the addition of graphite. After the initial running-in phase that lasted up to ~1400 s of testing, we continued to observe the formation and separation of build-ups from the sample surface; however, the dynamics of this process were clearly lower than in the case of sample K10.0. In the final phase of the tribological test of sample K10.2, from about 6000 s, we saw increasingly rare and smaller volume build-ups on the counter-sample.

The fluctuation of the vertical position of the counter-sample pin also shows a less dynamic character than in the case of the composite without graphite addition (Figure 7).

Figure 8 shows the course of the change in the friction coefficient μ and the course of the change in the vertical position of the steel pin during the tribological test with friction without lubrication of the K10.5 sample. In the case of this sample, the 5% addition of graphite to the composite matrix caused a further decrease in the dynamics of the mixed, abrasive–adhesive character of the tribological wear. After the initial running-in phase (from ~1400 s of the test), the formation and detachment of build-ups from the sample occurred less frequently than in the case of the K10.2 sample and much less frequently than in the case of the K10.0 sample. The fluctuation of the vertical position of the counter-sample pin occurred only at the moment when there was a clear growth and detachment of build-ups, e.g., 4000 s, 5420 s, 6100 s, and 7200 s of the test in Figure 6.

Figure 9 shows the course of the change in the friction coefficient μ and the course of the change in the vertical position of the steel pin during the tribological test with friction without the participation of lubricants of the K10.10 sample. In the case of this sample, the 10% addition of graphite to the composite matrix caused a complete change in the nature of the tribological wear. We were dealing with abrasive wear without the occurrence of an adhesive wear mechanism (occurring during the testing of samples K10.0, K10.2, and K10.5). We did not observe the phenomenon of formation and separation of adhesive build-ups on the K10.10 sample (Figure 4d). The addition of 10% graphite to the composite matrix acts as a lubricant in the case of this tribological pair.

After the initial phase of the test, i.e., running-in, which lasted up to ~1400 s, the fluctuation of the vertical position of the counter-sample pin virtually disappeared and the friction coefficient stabilized and increased slightly at a constant load (Figure 9).

Table 3 presents the results of the tribological tests, where all the obtained parameters are compared. The friction coefficients for individual samples were determined for two separate phases: phase I (running-in phase) and the actual test. For all samples, the end of phase I occurred around 1400 s of testing. In Figure 5, Figure 7, Figure 8 and Figure 9, with the initial 300 s cut off to reach a force of 30 N, this point falls around 1100 s.

## 4. Conclusions

The results of the comparative tribological tests of polyester–glass composite samples with the addition of recyclate and nanoadditive graphite powder made manually in friction without lubrication with a 6 mm diameter counter-sample made of 416 SS (X12Cr13) steel under the action of a normal force of N = 30 N for 7200 s are summarized in Table 3.

The tests revealed that this type of composite with recyclate without the addition of graphite, in friction without lubrication with a steel counter-sample, is characterized by a mixed abrasive–adhesive type of tribological wear. The polyester–glass composite with recyclate in the form of particles with a fraction of <0.074 mm added to the matrix in the friction pair with steel shows significant fluctuations of the friction coefficient μ during the entire test (μ in the range of 0.34–0.56). The addition of 2% and 5% graphite powder with a particle diameter of <0.074 mm to the composite matrix (in mass terms) gradually changes the nature of the tribological wear in friction without lubrication in a pair with a steel counter-sample. The addition of 10% graphite powder to the composite matrix completely changes the nature of the tribological wear of the material in a pair with a steel counter-sample in friction without lubrication. In the case of sample K10.10, after the running-in period, we see a stabilization of the friction coefficient at a relatively low level from 0.32 to 0.34. At the same time, during the tribological test of sample K10.10, the vertical fluctuation of the counter-sample disappears. The addition of 10% graphite acts in the friction pair as a lubricant additive, considering the above results and the fact that the K coefficient (mass wear rate) of sample K10.10 increased in relation to sample K10.5 and the negative change (the counter-sample sank into sample K10.10). It is noticeable that in the case of the 5% addition of graphite in the composite, the strength parameters obtained are the closest to the base material (without the addition of graphite). This is also reflected in the obtained friction coefficient. With an increase in percent graphite, it is lower. In the case of 10% graphite content, the smallest scatter in the obtained parameters is obtained. It seems advisable to carry out complementary tribological tests for a new material with the addition of graphite in the range of 5 to 10% and correlate these results with samples without recyclate addition and with only graphite addition. By analyzing the friction coefficient curves of all tested samples, it can be clearly seen that a 10% addition of graphite allows for a significant reduction in the friction wear of the material. These results will determine the further direction of research toward industrial applications of the material, e.g., on the floors of cargo spaces of trucks, railway freight cars, or the holds of vessels.

## Figures and Tables

**Table 1:** Percent (%) content of subsequent components included in the samples prepared for tribological testing.

Indication	% Content of Resin	% Content of Glass Mat	% Content of Polyester–Glass Recyclate	% Content of Graphite
K10.0	65	25	10	0
K10.2	63	23	10	2
K10.5	64	21	10	5
K10.10	66	14	10	10

**Table 2:** Strength parameters of the tested composite materials [22].

Indication	UTS [MPa]	ε [%]	E [MPa]
K10.0	113.73	2.04	7962
K10.2	87.72	1.89	6971
K10.5	100.9	2.08	7237

Where: UTS—ultimate tensile strength [MPa]; ε—maximum strain [%]; E—Young modulus [MPa].

**Table 3:** Summary of the results obtained.

Indication	µ of the Running-in Phase	µ	Sample Weight Before Testing/Sample Weight After Testing	Change in Sample Mass in Tribological Test		K the Rate of Mass Consumption of the Sample		Change in the Vertical Position of the Tribometer Pin	
				AVG	SD	AVG	SD	AVG	SD
	[-]	[-]	g	mg		10^−4^ mg/m		μm	
K10.0	0.36–0.62	0.34–0.56	5.57430 5.55690	17.4	0.7	106.89	3.48	−3.6	0.49
K10.2	0.26–0.54	0.28–0.46	9.06315 9.05840	4.75	0.33	29.18	2.01	+7.2	0.37
K10.5	0.46–0.56	0.28–0.52	9.41375 9.41310	0.65	0.08	3.99	0.17	+7.4	0.24
K10.10	0.52–0.56	0.32–0.34	7.43710 7.43280	4.30	0.21	26.42	1.22	−4.0	0.33

Where: AVG—average value; SD—standard deviation.

## Data Availability

The data presented in this study are available upon request from the corresponding author. The data are not publicly available due to large quantities.
